# Designing an effective dissolution test for bilayer tablets tailored for optimal melatonin release in sleep disorder management

**DOI:** 10.3389/fnut.2024.1394330

**Published:** 2024-05-06

**Authors:** Rebecca Bassetto, Emanuele Amadio, Francesco Ciampanelli, Stefano Perin, Pietro Ilari, Paolo Gaballo, Martina Callegari, Sara Feltrin, Jacopo Gobbo, Samuele Zanatta, Walter Bertin

**Affiliations:** Labomar SpA, Treviso, Italy

**Keywords:** dissolution, SIFC system, biorelevant media, melatonin, bioaccessibility, modulated release

## Abstract

This project aims to investigate the release performance of bilayer tablet (BL-Tablet) designed with both fast and slow-release technology, targeting sleep disorders. The tablet incorporates Melatonin, extracts of *Eschscholzia californica* and *Melissa officinalis*. In order to validate the effectiveness of the extended-release profile, an advanced dissolution test was herein proposed. This new method utilizes biorelevant intestinal fluid media and incorporates a stomach-to-intestine fluid changing (SIFC) system. To demonstrate the advantages of employing this method for assessing the controlled release profile of active ingredients, the dissolution results were compared with those obtained using the conventional EU Pharmacopoeia approach. Furthermore, the comparative analysis was extended to include a monolayer tablet version (ML-Tablet) lacking the slow-release technology. Technological characterization and bioaccessibility studies, including intestinal permeability test, were conducted as well to assess the pharmacological performance and bioavailability of active ingredients. The dissolution data recovered revealed that the two dissolution methods did not exhibit any significant differences in the release of ML-Tablet’s. However, the dissolution profile of the BL-Tablet exhibited notable differences between the two methods particularly when assessing the behavior of the slow-release layer. In this scenario, both methods initially exhibited a similar release pattern within the first approximately 0.5 h, driven by the fast-release layer of the tablet. Following this, distinct gradual and sustained releases were observed, spanning 2.5 h for the EU Pharmacopoeia method and 8 h for the new SIFC-biorelevant dissolution method, respectively. Overall, the novel method demonstrated a substantial improvement compared to conventional EU Pharmacopoeia test in evaluating the performance of a controlled slow-release technology. Remarkably, the prolonged release technology did not have an adverse impact on melatonin intestinal absorption, and, consequently, maintaining its potential bioavailability of around 78%. Concluding, this research provides valuable insights into how the innovative dissolution test can assist formulators in developing controlled release formulations.

## 1 Introduction

A significant portion of the population over the age of 65, potentially up to 50%, suffers from sleep difficulties. These sleep disorders, including night-time insomnia, are linked to a range of negative consequences. These involve heightened daytime sleepiness, reduced motor and cognitive performance, diminished workplace productivity, and an increased risk of accidents (1, 2). The sleep disturbances in older adults have been frequently linked to the reduction of melatonin production which occurs with the advance of age. From a biochemical view, melatonin hormone is synthesized by the pineal gland and primarily released during nighttime (3). The hypothalamus plays a crucial role in ensuring a high concentration of melatonin during the night and an extremely low synthesis during the daytime. Melatonin exerts its physiological actions through a family of specific, high-affinity cell membrane receptors that are G-protein-coupled. These receptors mediate melatonin’s effects on various biological processes, including sleep-wake cycles, body temperature regulation, and other circadian rhythm related activities (4). As a result, many individuals with occasional sleep difficulties turn to self-medication using melatonin-based supplements (5). Therefore, the utilization of melatonin as an active ingredient in dietary supplements, especially for the elderly population, has received substantial attention in recent years. Consequently, the field has been subject to regulatory oversight to ensure the safety and effectiveness of these products.

Melatonin is permitted as an active ingredient for food supplements in various European countries, each having distinct regulations regarding the recommended dosage. The authorized range typically spans from 0.28 mg to 2 mg of melatonin per day, as specified in ANSES Opinion Request No. 2016-SA-0209 from 2018 (6). Additionally, EFSA has issued a favorable opinion for two claims relating to the presence of melatonin in foodstuffs (Commission Regulation (EU) No. 432/2012):

- “Melatonin contributes to the alleviation of subjective feelings of jet lag,” for food which contains at least 0.5 mg of melatonin per quantified portion.
- “Melatonin contributes to the reduction of time taken to fall asleep,” for food which contains 1 mg of melatonin per quantified portion.

From a medical perspective, the primary route of administration for exogenous melatonin is typically oral. The key factor that significantly impacts the clinical effectiveness of melatonin is its short half-life: the time taken by the body to eliminate half of the drug dose for melatonin varies from 32 min (for a dose of 2 mg) to 126 min (for a dose of 4 mg) (7, 8). The short half-life is a crucial consideration when determining the timing and dosage of melatonin supplementation to achieve the desired therapeutic effects. Thus, it is necessary to maintain melatonin’s concentration for a long time to mimic its physiological release, especially in the context of insomnia treatment. It’s worth noting that a common concern associated with prolonged melatonin levels is the risk of daytime sleepiness. Some studies using prolonged-release melatonin with doses higher than 1 mg have not observed any next-day carryover effect (9, 10). Nevertheless, it remains crucial to create a modified formulation that ensures the sustained release of melatonin throughout the night, effectively managing sleep disorders without inducing unwanted daytime drowsiness (11).

During the last decades, a huge number of modified release systems have been developed, as they display several advantages over conventional systems used in therapeutics (12–15). One of the most common methods involves the use of various excipients during tablet manufacturing to enable controlled release (16). These excipients usually involve hydroxypropylmethylcellulose, polyvinylpyrrolidone, and sodium alginate in various molecular weights or forms (5). Modifying drug release is a common practice in the design of dosage forms which can be achieved through the application of functional coatings. Two types of modified-release dosage forms are described by the USP, those that are enteric-coated and those that are extended-release. Functional coatings, designed to prolong drug release over an extended period or to reduce the drug regimen, are commonly referred sustained- or extended-release coatings (17, 18). As an alternative option, multilayer tablets can be effectively tailored to offer both rapid and gradual release profiles based on the choice of excipients (18). One layer can provide immediate release, while another layer can deliver a sustained release, effectively acting as a dosage form able to maintain stable levels of active ingredient during an extended time (19–22).

The primary objective of this work is to investigate the release performance of a bilayer tablet (BL-Tablet) designed with both fast and slow-release technology, targeting sleep disorders. This formulation was designed to maintain melatonin concentration throughout the entire night, ultimately enhancing its therapeutic effectiveness in addressing sleep disorders. Moreover, the designed BL-Tablet not only contains melatonin but also integrates two additional active components: extracts of *Eschscholzia californica* and *Melissa officinalis* used in the fast and slow layer, respectively. California poppy (vulgar name of *Eschscholzia californica* Cham.), recognized for its sedative properties, plays a crucial role in amplifying the tablet’s ability to exert soporific effects and to improve sleep quality (23). On the other hand, Lemon balm (vulgar name of *Melissa officinalis* L.) known for its calming effects, significantly contributes to support mental relaxation and to aid sleep (24, 25). *Melissa officinalis* has been selected as an additional botanical ingredient in the slow-release layer to assist the sleep-inducing effect of melatonin; while *Eschscholzia californica* has been decided to be released fast taking into consideration it would have a relaxing action, without negative effects on daytime sedation. Overall, the fast and slow-release mechanism of melatonin, coupled with the calming and sedative effects of the additional active natural substances, creates a well-rounded and effective solution for those in need of faster sleep onset and better sleep quality.

In order to validate the effectiveness of the extended-release profile, within an environment that closely simulates physiological conditions, an advanced dissolution test was optimized. This new method utilizes biorelevant intestinal fluid media and incorporates a stomach-to-intestine fluid changing (SIFC) system. This test allowed for precise control of the release time profile under conditions that closely mimic the human body in case of fasted condition’s assumption of the melatonin tablet in accordance with the recommended instructions for consuming the nutraceutical product. Furthermore, to demonstrate the advantages of employing this method for assessing the controlled release profile of active ingredients, the dissolution results were compared with those obtained using the conventional EU Pharmacopoeia approach. The overall performance of BL-Tablet formulations was compared with that of a monolayer melatonin tablet that did not contain slow-release technology (ML-Tablet). The BL-Tablet formulations under study were subjected to different technological trials to determine tablet dimensions and organoleptic characteristics, as well as the uniformity of mass, hardness, disintegration, dissolution, and bioaccessibility. Moreover, permeability study over gastric biomimetic membrane was conducted as well to assess the pharmacological performance and bioaccessibility of the released melatonin.

## 2 Materials and methods

### 2.1 Materials and reagents

The reference standard of melatonin (99% purity) was obtained from Merck (Supelco PHR1767, Darmstadt, Germany). Water (HPLC grade), Methanol (HPLC grade) and Acetonitrile (HPLC grade) were purchased from Carlo Erba Reagents (Cornaredo, Milan, Italy). Polymeric dialysis-like supports were purchased from Millipore^®^ (Mixed Cellulose Esters VCWP02500, 0.1 μm × 25 mm, white plain; New York, NY, United States). The lipid phase used for the impregnation of the porous dialysis-like supports consisted of Lipoid^®^ E80 by Lipoid (Ludwigshafen, Germany), cholesterol and *n*-octanol were provided by Sigma-Aldrich (product number 8209311000, Milan, Italy). All the other chemicals were purchased from Sigma-Aldrich (Milan, Italy). 3F powder to make Fasted State Simulated Gastric Fluid (FaSSGF) and Fasted State Simulated Intestinal Fluid (FaSSIF) were purchased from Biorelevant (London, United Kingdom). BL-Tablet and ML-Tablet were produced by Labomar S.p.A.

### 2.2 Design and preparation of bi- and mono-layered tablets (BL- and ML-Tablet)

The BL-Tablet designed for extended-release melatonin was prepared by Labomar S.p.A. Following the standard procedure (so called Nutralayer^™^, a patented technology of Labomar), the ingredients contained in each layer were weighed and homogeneously mixed. The powders were loaded into a rotary tablet press (PZ-Tre, B&D Italia s.r.l.) and then any single layers were compacted into a matrix following a compression to produce the bilayer tablet. Using the same process, the ML-Tablet was produced, but without any distinction between the layers. Subsequently, the BL and ML-Tablets were blister-packed. Each ML and BL-Tablets contain a total amount of 1 mg of melatonin with as an approximate total weight of 1,200 mg. In the case of BL-Tablet each layers contain 0.5 mg of melatonin. These tablets underwent a comprehensive technological assessment covering various key parameters, weight, thickness, hardness, friability, abrasion, aW, LOD and disintegration. Friability, abrasion, aW, LOD were performed in accordance with USP (26–29). The disintegration assay was conducted in accordance with the standards outlined in the European Pharmacopeia 10.0 (30), using Charles Ischi AG disintegration tester (Zuchwill, Switzerland). The tablets were placed on a perforated plate and immersed in an 800 mL medium (HCl 0.1 M, pH1.0) at a controlled temperature of 37 ± 0.1°C. The top of the testing apparatus was sealed with a glass plate to maintain consistent testing conditions. The disintegration assay was considered complete after 30 min. Once the bilayer tablets passed the technological assessment and disintegration testing, stability studies were initiated following the guidelines provided in ICH Q1 (R2) (31). These stability studies aim to evaluate the long-term integrity and effectiveness of the tablets under various environmental conditions, including exposure to different temperatures and humidity levels. Detailed protocols and procedures, in accordance with ICH guidelines, were followed to assess the tablets’ stability over time.

### 2.3 Preparation of dissolution media

#### 2.3.1 EU Ph media

All the media were prepared in accordance with EU Pharmacopoeia. In details, HCl 0.1 M (pH 1.0) were prepared and used for the gastric step, instead for the small intestine step a phosphate buffer solution was prepared with 0.2 M potassium dihydrogen phosphate and 0.2 M sodium hydroxide in water to reach a pH6.8 ± 0.05 (32, 33).

#### 2.3.2 Biorelevant media

The composition of FaSSGF was prepared in accordance with biorelevant.com Ltd. (34). Pre-FaSSIF was prepared to finally achieve pH 6.5 and the concentrations specified by biorelevant.com Ltd., after the addition to FaSSGF.

FaSSIF, the ultimate solution, was achieved by combining the previously prepared FaSSGF and pre-FaSSIF compositions (Table 1).

**Table 1:** Composition of biorelevant media used to simulate fast state condition in gastrointestinal and intestinal tract.

Component	FaSSGF	pre-FaSSIF	FaSSIF
Taurocholate	0.08 mM	8.8 mM	3 mM
Phospholipids	0.02 mM	2.2 mM	0.75 mM
NaCl	34.19 mM	249.15 mM	105.84 mM
HCl 1 M	885.85 mM	—	—
NaOH	—	31.5 mM	10.5 mM
NaH_2_PO_4_	—	85.96 mM	28.65 mM
pH	1.6	10.5	6.5

### 2.4 Dissolution test

Two different dissolution test methods using USP apparatus 2 (paddle) (Electrolab TDT-08, Mumbai, India) were investigated: EU Pharmacopoeia method with compendial media and SIFC-biorelevant dissolution test, a more *in vivo* correlated alternative method.

#### 2.4.1 Delayed release solid dosage forms—EU Pharmacopoeia methods

The dissolution test in compendial media was carried out using USP apparatus 2 (paddle) (TDT-08, Electrolab, Mumbai, India).

The dissolution medium was 750 mL of filtered and degassed 0.1 M HCl for 2 h, followed by the addition of 250 mL phosphate buffer at a final pH of 6.8, maintained at 100 rpm and 37.0 ± 0.5°C. Samples were collected at specified time points (from 0 h for up to 8 h), filtered through a 0.45 μm filter, and analysed for melatonin content with a high-performance liquid chromatography (HPLC) (Vanquish, Thermo-scientific, United States).

#### 2.4.2 SIFC-biorelevant dissolution test

The optimized dissolution test was performed according to Takagi et al. (35) method with slight adjustments. The test was designed to simulate physiological conditions encountered during drug administration in humans under fasted conditions, with volumes adjusted to accommodate the use of the USP apparatus 2. Additionally, the number of tablets used were adjusted as well to ensure analytical sensitivity for determining the concentration of melatonin. In the first step, the USP apparatus 2 (paddle) contains the FaSSGF, which simulates the acidic environment of the stomach was used. After a specified time, in a second step, to the dissolution medium was added the FaSSIF, representing the pH environment of the small intestine. In more details, as outlined by Takagi et al. (35), the introduction of pre-FaSSIF with a pH of 10.5 to FaSSGF with a pH of 1.6 starts after a 1 min incubation period. This step is intended to replicate the swift pH transition observed in the gastrointestinal tract following oral drug administration under fasted state conditions. Following this initial incubation, a gradual addition of pre-FaSSIF into FaSSGF facilitates a pH transition from the stomach’s acidic environment (pH 1.6) to the typical pH range of 6–7 observed in the small intestine within approximately 10 min. During the dissolution test, samples are withdrawn at specific time intervals, and the drug concentration in the samples is analysed using appropriate analytical techniques. The obtained dissolution data from the new system is then used to construct a dissolution profile, which graphically represents the drug’s release rate over time.

**Figure 1:**
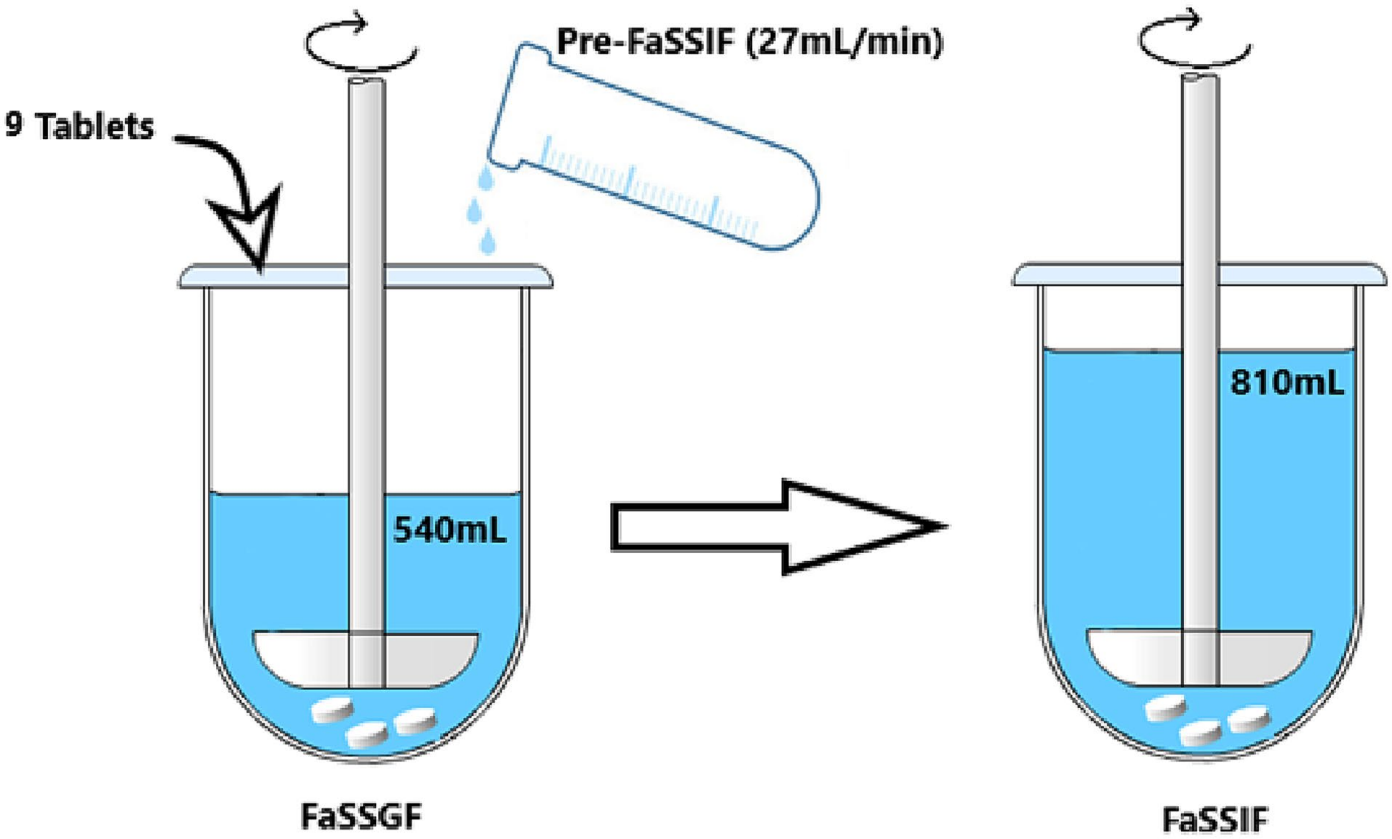
Schematic view of the experiment conditions for SIFC-biorelevant dissolution test.

In more details, to perform the dissolution test in simulated digestion condition (Figure 1), 9 tablets were added into 540 mL of FaSSGF. After 1 min of pre-incubation, 270 mL of pre-FaSSIF was added at a rate of 27 mL/min for 10 min, and the monitoring of the drug dissolution was started. Pre-FaSSIF was prepared to finally construct pH 6.5 after addition to 270 mL of FaSSGF, adjust the pH with NaOH 2 M or HCl 2 M. After completion of pre-FaSSIF infusion, drug dissolution was monitored up to the complete dissolution of the tablet, and 1 mL of solution was withdrawn at specific time.

### 2.5 Permeability studies

Permeability study was performed using Franz cell (Copley Scientific, United Kingdom) apparatus following the melatonin passage over a gastro-intestinal biomimetic membrane (36–40). The gastrointestinal biomimetic membrane was prepared by assembling a Mixed Cellulose Esters membrane with a mixture of phospholipids and cholesterol, as described in references (36, 41). In the donor, 3 mL of pre-filtered dissolution test solution (withdrawn after 8 h of dissolution test) was added and covered to prevent evaporation. Samples from receiving chamber were collected during 6 h and then analysed by HPLC (Vanquish, Thermo-Scientific, United States). At the end, the concentration in receiving chamber, the flux (g/s·cm^2^) and apparent permeability (cm/s) were determined. Additionally, at the end of the experiment, the solution recovered from the donor chamber was analysed to assess the remaining amount of melatonin. To determine the mass balance, the membrane was left overnight in 2 mL of ACN:H_2_O = 20:80% to extract the melatonin that was entrapped. The resulting solution was also analysed.

### 2.6 High-performance liquid chromatography assay

Melatonin (MLT) was quantified by high-performance liquid chromatography (HPLC-DAD). The analyses were conducted using a VANQUISH Core/Ultimate 3,000 system from Thermo Fisher Scientific (Waltham, Massachusetts, United States). This system consisted of a pump, autosampler, column oven, and diode array detector (DAD).

A reverse-phase column, specifically the Acclaim™ C18 column (150 mm × 4.6 mm dimensions, 5 μm particle size), also from Thermo Fisher Scientific, was utilized and maintained at a constant temperature of 30°C. For both standard solutions and samples, an injection volume of 20 μL was injected. The detector wavelength was set at 278 nm to monitor MLT.

The mobile phase employed in the chromatography consisted of a mixture of deionized water and acetonitrile, delivered at a flow rate of 1 mL/min using gradient elution. The total run time for the analysis was 23 min.

Calibration curve solutions were prepared using a MLT reference standard dissolved in a solvent composed of a 25:75 ratio of acetonitrile to water. Additionally, samples for specificity evaluation consisted of placebo solutions (fluid used for dissolution without MLT) and fluid used for dissolution spiked with 100% of analyte. It’s worth noting that all samples obtained from both dissolution and permeability tests were analysed as-is without further manipulation, ensuring the integrity of the results. The calibration curve and typical chromatograms were reported in Supplementary Figures S1, S2.

### 2.7 Data analysis

All experiments were performed at least in tripled independent replicates for each set of conditions. The reported values are presented as means along with the standard deviation (SD) to indicate the variation from the average value and represented as mean ± SD. In order to determine if there is a significant difference between the means of two groups, a statistical test (*t*-test) was conducted to provide evidence. A *t*-value higher than 0.05 (*p* > 0.05) indicates that the groups are different while a *t*-value lower than 0.05 (*p* < 0.05) indicates that the groups are similar. The data from HPLC were acquired using Chromeleon 7 software (Thermo Fisher Scientific, Waltham, Massachusetts, United States). All the collected data underwent processing and statistical analysis using Microsoft Excel (Microsoft, Redmond, Washington, United States).

## 3 Results and discussion

### 3.1 BL-Tablet: characterization and stability features

Within the scope of the work, this section describes the design and development of the BL-Tablet. It covers the rational choice of the excipients and the slow-release technology, and the characterization carried out for guaranteeing the consistency and quality of the formulation. This includes an examination of technological parameters like weight, thickness, hardness, friability, abrasion, aW, LOD, and disintegration. A stability study for the BL-Tablet was conducted as well to assess its conformity over the course of its shelf life.

Regarding the design, from a theoretical point of view, the bilayer tablet should be structured to achieve a controlled release of melatonin that serves a dual purpose: delivering both fast and slow melatonin release. This outcome can be made possible through the inclusion of specific excipients and active substances in each layer of the tablet. (i) The fast-release behavior is ensured using cross-linked sodium carboxymethylcellulose E 468 (Roquette, Lestrem, Francia) excipient into the fast-layer formulation. This excipient it is known to facilitate the rapid disintegration of the tablet upon ingestion (42). As a result, it enables the quick and immediate release of melatonin. This rapid release is beneficial for individuals seeking prompt sleep-inducing effects and a shorter onset time for melatonin’s action. (ii) Conversely, the slow-release layer of the tablet is designed to provide sustained, controlled release of melatonin over an extended period. This layer incorporates retarding agents such as a combination of sodium alginate E 401 (Idealfoods S.p.A., Milan, Italy) and hydroxypropylmethylcellulose E 464 (Shin-Etsu Chemical Co., Ltd., Niigata, Japan). These excipients work in synergy to modulate the release of melatonin, ensuring its gradual delivery over a more prolonged duration. In addition to melatonin, BL-Tablet contains natural extracts of *Eschscholzia California* and *Melissa officinalis* delivered in the fast and slow layer, respectively. The combination of melatonin and botanicals delivered with a modulated release technology will offer users a comprehensive and unique natural approach promoting a deeper and more restful sleep experience.

**Figure 2:**
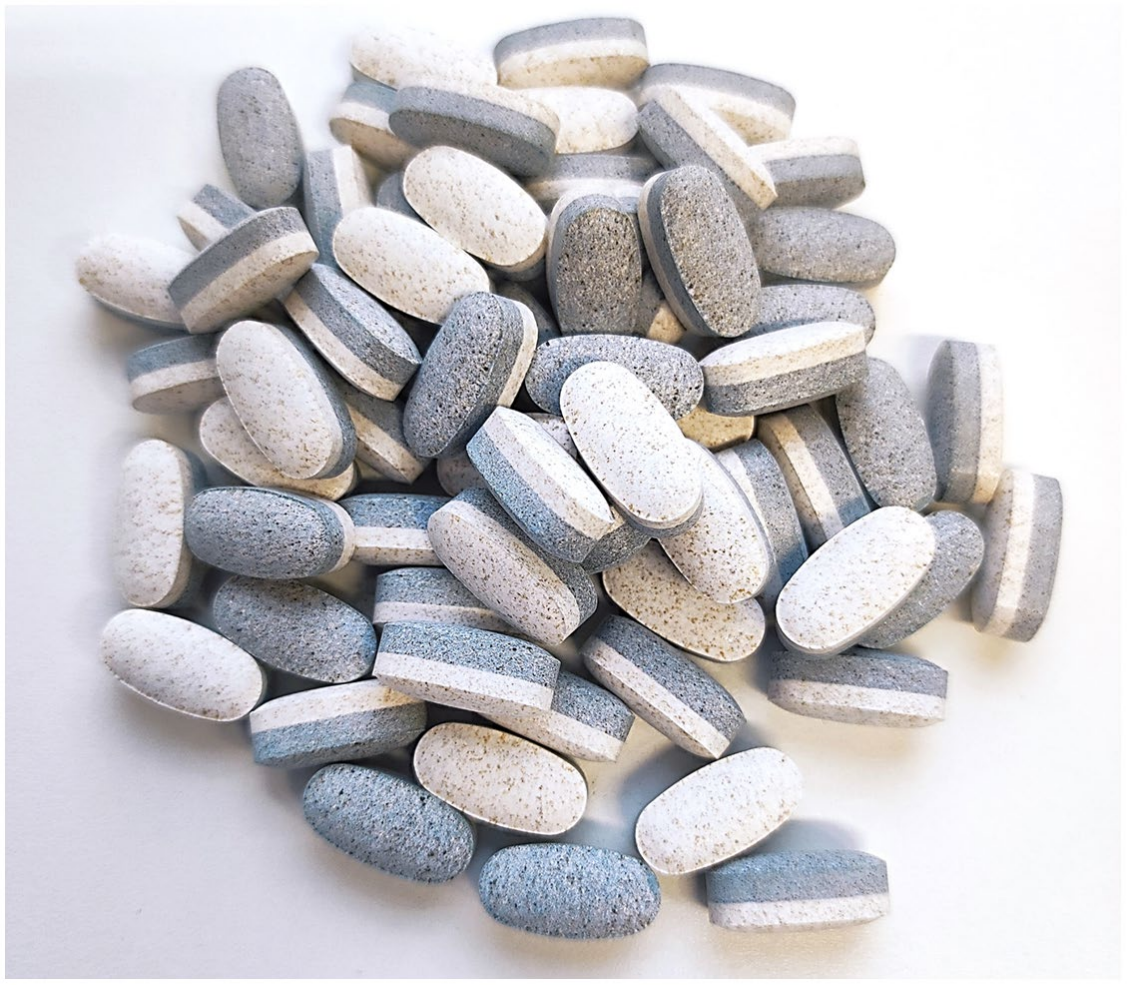
BL-Tablet produced by direct compression.

For the reason of comparison, ML-Tablet was formulated using the same components, with the exclusion of retarding agents neither layers division was designed as well. With the defined formulations, the BL- and ML-Tablets (Figure 2) were manufactured by direct compression of the corresponding powder blends.

After the production, the BL-Tablet underwent a rigorous assessment involving several key parameters to ensure its quality and performance. These parameters include weight, thickness, hardness, friability, abrasion, aW, LOD and disintegration. The tablet’s weight provides an essential metric for quality control and dosing accuracy, ensuring that each tablet contains the intended quantity of active ingredients, promoting therapeutic effectiveness. Tablet thickness is another crucial dimension that contributes to patient compliance. Ensuring consistent tablet thickness helps maintain a standardized dosage. Tablet hardness is a measure of the force required to break the tablet, this parameter is important to ensure that the tablet maintains its structural integrity during handling, packaging, and transit. The evaluation of tablet friability is crucial to determine the tablet’s resistance to abrasion or chipping during handling and transportation; low friability indicates that the tablet is less likely to break or crumble, ensuring product integrity and preventing potential issues related to tablet breakage, while abrasion is the mass loss from powder erosion as the result of tablets rubbing together in coater drums or during packaging, handling or shipping. Water activity (aW) is a measure of the energy status of water in a product. This value indicates how tightly water is bound structurally or chemically, within a substance. The loss on drying test (LOD) is a method to measure the loss in mass of the tablet, when dried in specific conditions. The disintegration test determines whether tablets, capsules, or granules disintegrate within the prescribed time when placed in a liquid medium at specific experimental conditions (Table 2).

**Table 2:** BL-Tablet’s characterization data.

Parameter	Value	Acceptability range
Weight	1,045–1,155 mg	—
Thickness	7.2–7.4 mm	—
Hardness	22–26 kg	—
Friability	0.29% (4 min)	<1% (27)
Abrasion	0.19% (4 min)	<1% (28)
aW	0.184 (27.1°C)	<0.6 (29)
LOD	1.86% (5.036 g)	<5% (26)
Disintegration	<30 min, fast layer	—

Following the technical characterization of the BL-Tablet stability study was executed. These comprehensive studies are essential in ensuring BL-Tablet becoming a market-ready nutraceutical product. The purpose of the stability studies is to provide valuable evidence regarding how the quality and effectiveness of a product evolve over time when exposed to various environmental factors. The primary factors under consideration typically encompass temperature, humidity, and light exposure. In-depth stability testing of the BL-Tablets was conducted, with a focus on long-term stability extending over a substantial 36-month period covering environmental conditions of both Zone IVA (30 ± 2°C, 65 ± 5% humidity) and Zone II (25 ± 2°C, 60% ± 5% humidity) (31). Throughout the stability testing period, the BL-Tablets consistently retained their appearance, preserving both their visual characteristics and structural integrity. Additionally, the content of melatonin (utilized as a quantified marker) remained unchanged exhibiting a dosage of 1 mg ± 20% and 1 mg ± 20% after 36 months of stability Zone IVA and Zone II, respectively, (see Supplementary Figure S3).

In summary, the collected data highlight the conformity of all technological parameters for the BL-Tablets, aligning with standard pharmaceutical parameters (43) and expertise gained for similar tablets previously industrialized in the field. Additionally, the formulation of BL-Tablets ensures a substantial 36-month period of shelf-life.

### 3.2 Bioaccessibility studies

In bioaccessibility studies, the primary objective is to assess the extent to which nutraceuticals are released from the test samples and dissolved in the simulated gastrointestinal fluids. The secondary goal is to quantify the amount that can be absorbed and made bioavailable for the human body. This process is crucial in understanding the efficacy of the nutraceutical product and thus in determining the amount of phyto-actives that can be effectively utilized by the body (44). In these studies, melatonin was utilized as an indicative marker of the entire mixture.

In more details, to determine the release of melatonin, two different dissolution tests were conducted. Additionally, an absorption test over a gastro-intestinal biomimetic membrane was performed to assess the bioavailability of the released melatonin. The dissolution and absorption performances were then compared between BL-Tablet and ML-Tablet formulations. This comparative analysis aims to provide valuable insights into the characteristics and potential differences between these formulations.

#### 3.2.1 Dissolution profiles and comparison between formulations

As previously mentioned, the initial step in a bioaccessibility study is the dissolution test. This test provides crucial information about the degree to which melatonin is liberated from the tablet matrix and how efficiently it dissolves in body fluids. Setting the correct operative conditions and selecting the appropriate dissolution fluids to closely simulate the bio-relevant body environment are crucial factors to consider when conducting dissolution studies. These factors play a pivotal role in ensuring that the study accurately reflects how a tablet formulation behaves in the human body (45, 46).

Various apparatuses were developed, and different protocols were established to ensure consistent and reliable results of a dissolution test in the last decades (47–51). In this regard, Pharmacopeias—including United States Pharmacopeia (USP) and European Pharmacopoeia (Ph. Eur.)—have played an essential role in establishing standards and guidelines for the pharmaceutical and related industries and in providing essential *in vitro* data on the release of active components (32, 52). The dissolution test conditions, such as the choice of dissolution media, apparatus, and testing parameters, are standardized and defined in pharmacopeial monographs to ensure uniformity and reproducibility of results across different laboratories and manufacturers. It serves as a critical quality control tool for the pharmaceutical industry to monitor batch-to-batch consistency and compliance with regulatory standards.

Nevertheless, it’s important to note that the dissolution tests prescribed in Pharmacopeia may not fully replicate the intricate and dynamic *in vivo* conditions found within the gastrointestinal (GI) tract (53). After oral administration, drugs encounter the acidic environment of the stomach, followed by the more alkaline environment of the small intestine. The pH changes affect drug solubility, dissolution, and absorption (54).

Therefore, the development of an optimized SIFC-biorelevant dissolution test that better replicates the *in vivo* passage of the product through the gastrointestinal system becomes essential. Indeed, the development of such a test will be a valuable tool for formulators in fine-tuning tablet formulations and refining manufacturing processes at the early stages of product design and development to achieve the desired pharmacological activity (55, 56).

For this reason, the dissolution profiles were assessed using both the Pharmacopoeia-prescribed method and an optimized SIFC-biorelevant dissolution test using USP dissolution test apparatus 2. This test has been specifically designed to simulate the consumption of the melatonin tablet under the conditions of fasted condition (i.e., empty stomach), in accordance with the recommended instructions for consuming these nutraceutical products.

In deeper, this system aims to represent the pH shift of human GI fluid in the fasted state using a single chamber. The optimized test incorporates the use of biorelevant fluid media in combination with the SIFC (stomach-to-intestine fluid changing) system (35). Biorelevant media are solutions designed to mimic specific regions of the GI tract such as gastric FaSSGF (Fasted State Simulated Gastric Fluid) and intestinal FaSSIF (Fasted State Simulated Intestinal Fluid) (57, 58). The SIFC system is designed to simulate the dynamic changes in pH and composition of gastrointestinal fluid that occur as orally administered drugs transition from the stomach to the small intestine.

Once the appropriate experimental conditions have been identified, both the Pharmacopeia-prescribed method and the new SIFC-biorelevant dissolution test were established. These tests were conducted on BL-Tablet and ML-Tablets for the purpose of comparison with a focus on their dissolution profiles, monitoring melatonin concentration over an 8 h duration.

**Figure 3:**
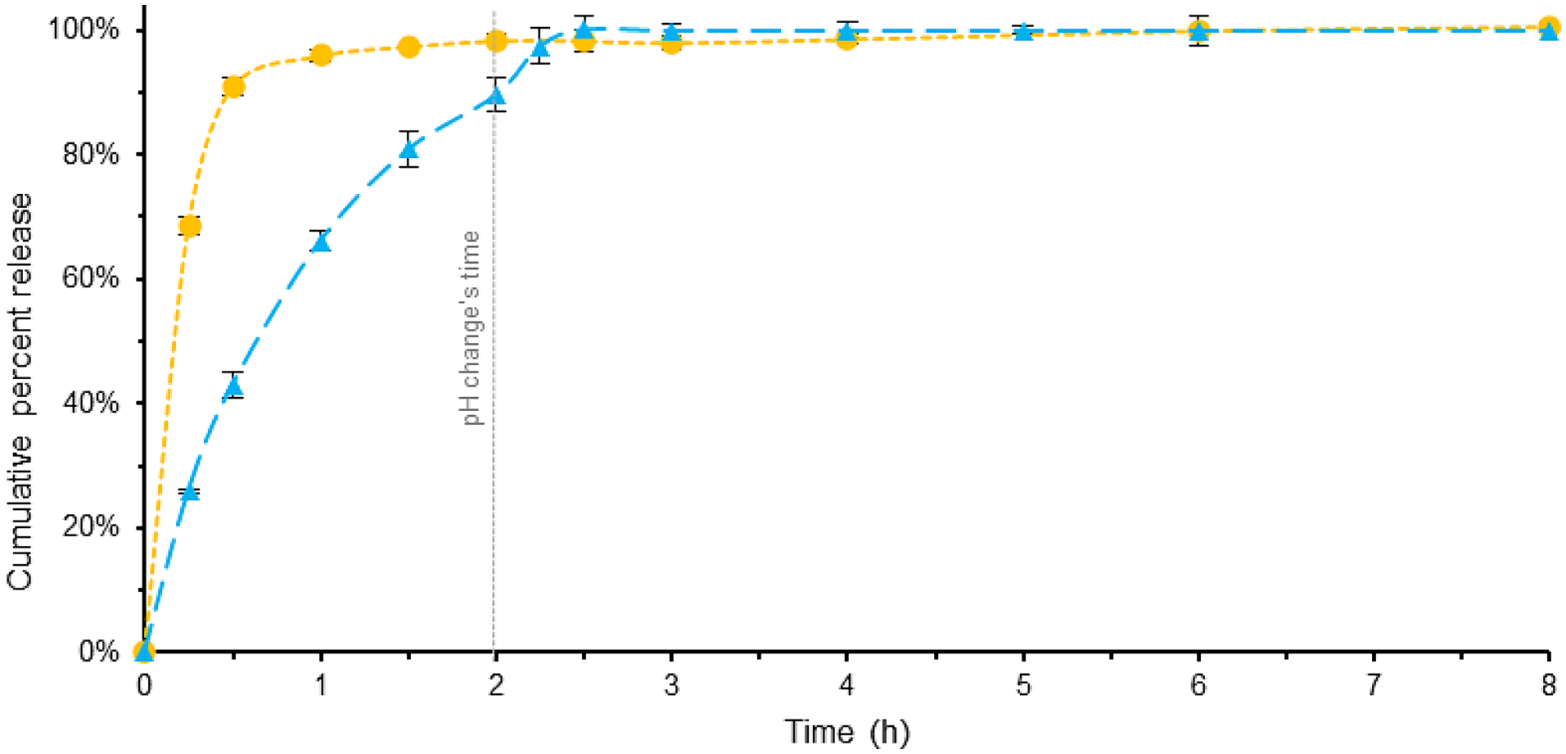
Comparison of melatonin dissolution in a BL-Tablet (blue) and a ML-Tablet (yellow), using EU Pharmacopoeia dissolution method. pH changes time (gray). Error bars represent standard deviation.

In details, Figure 3 (see also Supplementary Tables S1, S3) showed a comparison between these tablets using the Pharmacopeia method. As shown in Figure 3, the PH changes occurs at 2 h. Specifically, with the addition of phosphate buffer to the acidic dissolution medium, the pH undergoes a change from 1.2 to 6.8 (for a more comprehensive explanation, refer to the experimental section).

ML-Tablet exhibit a fast and complete dissolution within approximately 1.5 h.

In contrast, the BL-Tablet exhibited a slower and more gradual release profile. Approximately 43% of the melatonin was released within the initial 0.5 h, indicating a relatively slow onset of release. Complete dissolution of the tablet was achieved in approximately 2.5 h, suggesting a sustained release pattern compared to the fast-release counterpart.

The area under the curve (AUC) provides information about the extent at which the active ingredient is released, and it is indicative of the release profile of the active ingredient over time. In this case, the AUC of the blue profile is lower than that of the yellow profile, indicating the slow-release behavior of the BL-Tablet.

**Figure 4:**
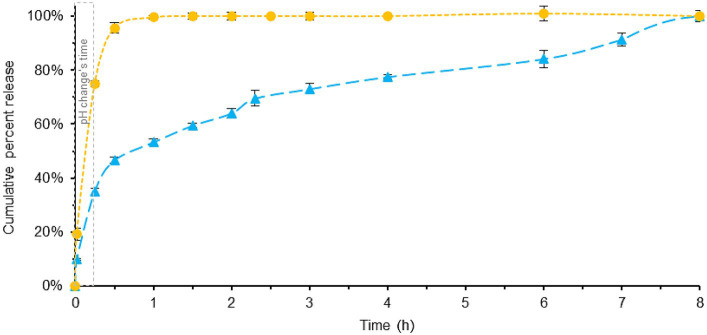
Comparison of melatonin dissolution in a BL-Tablet (blue) and a ML-Tablet (yellow), using SIFC biorelevant dissolution method. pH changes time (gray). Error bars represent standard deviation.

In Figure 4 (see also Supplementary Tables S2, S4), the dissolution profiles of the two melatonin tablet formulations are shown using the new SIFC-biorelevant dissolution test. As shown in Figure 3, the pH changes between 1–15 min. Specifically, with the addition of pre-FaSSIF to the acidic dissolution FaSSGF medium, the pH undergoes a change from 1.6 to 6.5 (for a more comprehensive explanation, refer to the experimental section).

The results reveal noticeable differences between the two formulations, and, when compared with the previously described Pharmacopeia dissolution profile, some interesting findings emerge.

In the case of ML-Tablet, there are no significant differences observed between the two dissolution tests (see Supplementary Figure S4). In both cases, the fast-release profile remains consistent, with melatonin being totally released in approximately 1.5 h. This suggests that the rapid-release characteristics of the tablet are not significantly influenced by the test method used, mainly because the substance is readily available for release and dissolution.

On the contrary, the dissolution profile for the BL-Tablet exhibits distinct behavior between the two tests. Both methods show very similar release pattern within the first 0.5 h (see Supplementary Figure S5), with around 43 and 47% of melatonin being released after 0.5 h for Pharmacopeia and SIFC-biorelevant dissolution method, respectively. This reasonably implies that the melatonin released in this initial phase predominantly belongs to the fast-release layer of the tablets. After the initial 0.5 h noticeable, differences between the two methods were instead observed. These variations are likely due to the slow-release layer’s response being significantly influenced by the specific testing conditions. Specifically, with the optimized method, an extended and gradual release of melatonin is observed, reaching total dissolution after 8 h. This contrasts with the Pharmacopeia’s dissolution method, which achieves near-total dissolution in approximately 2.5 h. Also in this case the area under the curve (AUC) demonstrates that BL-Tablets has a slower-release profile than ML-Tablet.

The disparities observed in the dissolution profiles of the BL-Tablet between the two testing methods can be attributed to a combination of factors. These include the utilization of more physiologically relevant conditions and the incorporation of a slow-release layer within the tablet’s formulation. Nevertheless, it’s worth noting the influence of the brief gastric step in the SIFC system and the variations in composition present in both testing systems. Despite the fact that comprehending the reasons is beyond the scope of the current study, these findings underscore the importance of employing biorelevant dissolution testing methods, especially when dealing with drug formulations designed with modified release profiles. By using conditions that closely simulate the physiological environment, SIFC biorelevant dissolution method provides a more accurate prediction of how the nutraceutical formulation -with modified release profiles-behaves *in vivo*.

### 3.3 Melatonin intestinal absorption as bioavailability prediction

The second objective of the bioaccessibility studies was to quantify the melatonin released from tablets capable of permeating and bioaccumulating in an intestinal membrane. This aimed to determine whether the diverse fast and slow-release technologies employed in the formulations could potentially impact the bioaccessibility of melatonin. This consideration is a crucial aspect in elucidating how the release mechanisms of the tablets might influence the absorption and availability of melatonin in the body, ultimately affecting their therapeutic efficacy in addressing sleep disorders (59). Permeability studies involve the measurement of how readily a substance, in this case, melatonin, can pass through biological barriers, such as the gastrointestinal mucosa (36, 60). The most common strategy for simulate the permeability of human gastrointestinal tract is represented by the use of *in vitro* models. Among these, the Franz-cell system is widely employed due to its simplicity, reproducibility, and cost-effectiveness (61).

**Figure 5:**
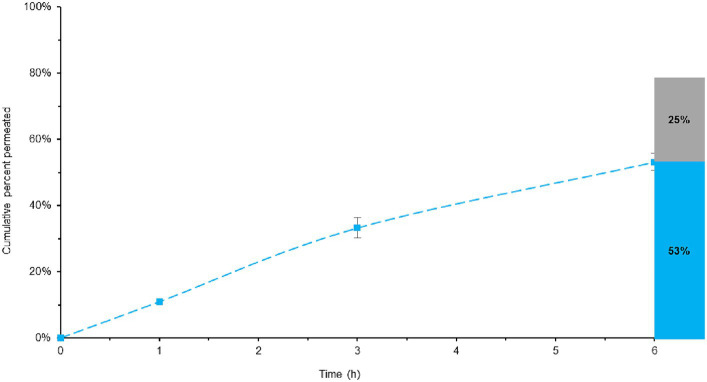
Total melatonin bioavailable in BL-Tablet, considering both the permeated and entrapped melatonin. Blue line represent the melatonin permeability overtime. The histogram illustrates the maximum quantity of potentially bioavailable melatonin, with the permeated melatonin shown in blue and the melatonin entrapped into the membrane in grey.

Based on the previously discussed dissolution data, particularly focusing on BL-Tablet formulation, it was observed that complete melatonin release occurred after 8 h when using the more physiologically relevant SIFC-biorelevant dissolution test. To better simulate the biological conditions, the resulting dissolution fluid containing 100% (to respect the target value) of melatonin was subjected to a permeability test. Furthermore, the data obtained were compared with a similar experiment carried out using the dissolution media of the ML-Tablet. The aim was to verify the efficacy of the BL-Tablet formulation and, thus, that the incorporation of retarding agents does not adversely affect absorption. In Figure 5 the data related to melatonin permeability for BL-Tablet are shown. The permeability test was conducted over a 6 h period. The amount of melatonin that permeated through the membrane during this time was determined by analyzing the receiving chamber solution by HPLC-DAD. Furthermore, upon the completion of the permeability test, the melatonin entrapped within the membrane was extracted and quantified. This comprehensive mass-balance approach allowed us to predict the melatonin’s bioavailability.

Remarkably, the results indicate that approximately 53% of melatonin permeated after prolong exposure (6 h) while 25% remain entrapped within the membrane, leading to the conclusion that about 78% of the introduced melatonin could be potentially bioavailable (Figure 5). This result reinforces the robust bioavailability of melatonin and imply that it can efficiently permeate the gastrointestinal membrane, ultimately becoming accessible for absorption within the body.

**Figure 6:**
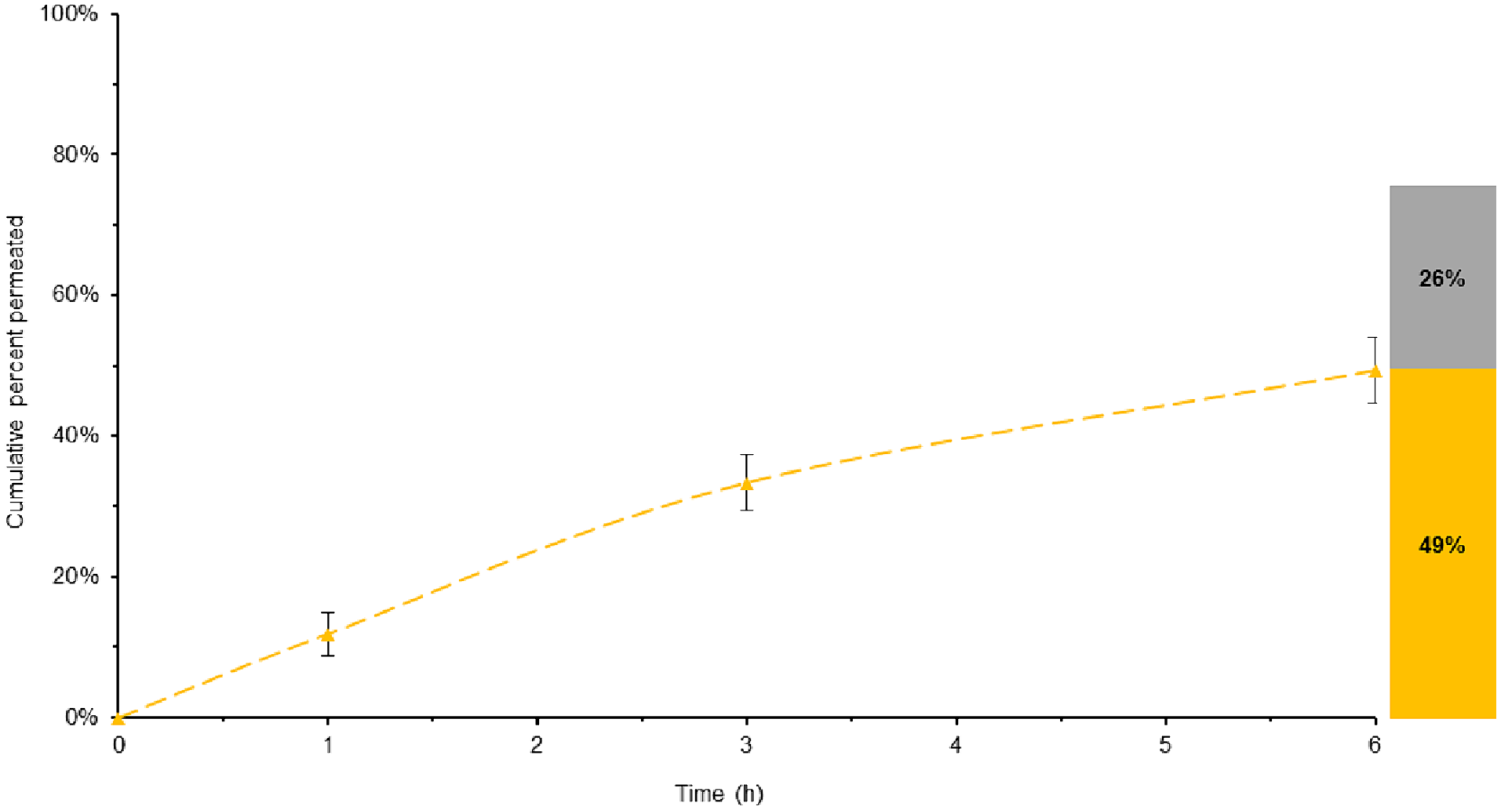
Total melatonin bioavailable in ML-Tablet, considering both the permeated and entrapped melatonin. Yellow lines represent the melatonin permeability overtime. The histogram illustrates the maximum quantity of potentially bioavailable melatonin, with the permeated melatonin shown in yellow and the melatonin entrapped into the membrane in grey.

In the assessment of BL-Tablet, a comparative evaluation of its permeability was conducted in comparison to ML-Tablet (Figure 6).

The data obtained from this comparison revealed no significant variations (*p* > 0.05) in bioaccessibility between the BL-Tablet and the ML-Tablet (see Supplementary Figure S6). These findings suggest that the incorporation of retarding agents into the BL-Tablet formulation does not exert a substantial influence on the absorption of melatonin.

## 4 Conclusion

The project successfully optimized a dissolution test, demonstrating its effectiveness in analyzing the release profile of BL-Tablets specifically designed with melatonin, extracts of *Eschscholzia californica* and *Melissa officinalis* to target sleep disorders. By incorporating biorelevant intestinal fluid media along with a stomach-to-intestine fluid changing (SIFC) system, the novel test achieves a more precise replication of the *in vivo* pathway, thereby giving a substantial advantage over conventional EU Pharmacopoeia methods. Indeed, the official pharmacopeia techniques commonly rely on simple aqueous buffers, which, while effective in simulating standard pH conditions in the stomach or small intestine, fall short in representing the *in vivo* situation that influence the release from the tested dosage form (62). Herein these two tests were employed to evaluate the release profile (using melatonin as a marker) of the designed BL-Tablets, and this was compared with the ML-Tablet, which lacks retarding agents. The dissolution data recovered indicated that the fast-release characteristics of the ML-Tablet were not significantly influenced by the test method used. This was primarily because melatonin in this formulation is readily available for release and dissolution. In contrast, the dissolution profile of the BL-Tablet exhibited notable differences between the two tests. Both methods initially showed a similar release pattern within the first approximately 0.5 h, thanks to the fast-release layer of the tablet. However, after the initial 0.5 h, significant variations emerged due to the behavior of the slow-release layer. A slow and gradual release was observed, ultimately reaching complete dissolution within a range of 2.5 to 8 h for the Pharmacopoeia and the new SIFC-biorelevant dissolution methods, respectively. In summary, the effectiveness of the BL-Tablet’s slow-release feature depends significantly on the chosen testing method. Despite variations in test outcomes, the BL-Tablet consistently delivers a prolonged and gradual release of melatonin, promising improved therapeutic benefits for those experiencing overnight sleep difficulties. In addition to the dissolution studies, absorption tests were conducted on an intestinal biomimetic membrane to assess melatonin’s bioavailability. The results show that around 53% of melatonin permeates the membrane, while 25% remains trapped within the membrane. This implies that approximately 78% of the introduced melatonin has the potential to be bioavailable. Importantly, the inclusion of retarding agents in the BL-Tablet’s formulation does not have an adverse impact on melatonin absorption. Overall, this research offers valuable insights into comparing the effectiveness of two different dissolution tests, highlighting the distinct release profiles of bilayer tablets featuring fast/slow-release technology. Furthermore, it underscores the importance of conducting *in vitro* tests in defining the appropriate formulation to achieve the desired nutraceutical effects of the product.

## Data availability statement

The raw data supporting the conclusions of this article will be made available by the authors, without undue reservation.

## Author contributions

RB: Data curation, Formal analysis, Investigation, Methodology, Software, Writing – original draft. EA: Conceptualization, Data curation, Formal analysis, Investigation, Methodology, Project administration, Software, Supervision, Validation, Visualization, Writing – original draft, Writing – review & editing. FC: Conceptualization, Supervision, Writing – original draft. PI: Writing – original draft. PG: Writing – original draft. MC: Writing – original draft. SF: Writing – original draft. JG: Writing – original draft. SZ: Supervision, Writing – review & editing. WB: Resources, Writing – review & editing. SP: Formal analysis, Writing – review & editing.

## References

[1] Carretero VJ Ramos E Segura-Chama P Hernández A Baraibar AM Álvarez-Merz I . Non-excitatory amino acids, melatonin, and free radicals: examining the role in stroke and aging. Antioxidants. (2023) 12:1844. doi: 10.3390/antiox12101844, PMID: 37891922 PMC10603966

[2] Haimov I Laudon M Zisapel N Souroujon M Nof D Shlitner A . Sleep disorders and melatonin rhythms in elderly people. BMJ. (1994) 309:167. doi: 10.1136/bmj.309.6948.167, PMID: 8044096 PMC2540689

[3] Skubis-Sikora A Sikora B Małysiak W Wieczorek P Czekaj P. Regulation of adipose-derived stem cell activity by melatonin receptors in terms of viability and osteogenic differentiation. Pharmaceuticals. (2023) 16:1236. doi: 10.3390/ph16091236, PMID: 37765045 PMC10535461

[4] Evans AT Vanden Brink H Lim JS Jarrett BY Lin AW Lujan ME . Overnight melatonin concentration and sleep quality are associated with the clinical features of polycystic ovary syndrome. Biomedicines. (2023) 11:2763. doi: 10.3390/biomedicines11102763, PMID: 37893137 PMC10604825

[5] Vlachou M Tragou K Siamidi A Kikionis S Chatzianagnostou AL Mitsopoulos A . Modified *in vitro* release of the chronobiotic hormone melatonin from matrix tablets based on the marine sulfated polysaccharide ulvan. J Drug Deliv Sci Technol. (2018) 44:41–8. doi: 10.1016/j.jddst.2017.11.019

[6] ANSES. ANSES’s opinion on the risks associated with the consumption of food supplements containing melatonin. Available at: https://www.anses.fr/en/content/ansess-opinion-risks-associated-consumption-food-supplements-containing-melatonin

[7] Harpsøe NG Andersen LPH Gögenur I Rosenberg J. Clinical pharmacokinetics of melatonin: a systematic review. Eur J Clin Pharmacol. (2015) 71:901–9. doi: 10.1007/s00228-015-1873-4, PMID: 26008214

[8] ANSES. Relatif Aux Risques Liés À La Consommation De Compléments Alimentaires Contenant De La Mélatonine. Avis l’Agence Natl sécurité Sanit l’alimentation, l’environnement du Trav. (2018) 33. Available at: https://www.anses.fr/fr/system/files/NUT2016SA0209; http://www.efsa.europa.eu/en/people/fpmembers%0Awww.anses.fr

[9] Wade AG Ford I Crawford G McConnachie A Nir T Laudon M . Nightly treatment of primary insomnia with prolonged release melatonin for 6 months: a randomized placebo controlled trial on age and endogenous melatonin as predictors of efficacy and safety. BMC Med. (2010) 8:51. doi: 10.1186/1741-7015-8-51, PMID: 20712869 PMC2933606

[10] Luthringer R Muzet M Zisapel N Staner L. The effect of prolonged-release melatonin on sleep measures and psychomotor performance in elderly patients with insomnia. Int Clin Psychopharmacol. (2009) 24:239–49. doi: 10.1097/YIC.0b013e32832e9b08, PMID: 19584739

[11] Vlachou M Siamidi A. Melatonin modified release formulations designed for sleep disorders In: Melatonin - Molecular Biology, Clinical and Pharmaceutical Approaches. UK: IntechOpen (2018). doi: 10.5772/intechopen.78337

[12] Okunlola A. Design of bilayer tablets using modified dioscorea starches as novel excipients for immediate and sustained release of aceclofenac sodium. Front Pharmacol. (2015) 5:294. doi: 10.3389/fphar.2014.00294, PMID: 25628566 PMC4290548

[13] Chung S Youn S Park B Lee S Kim C. The effectiveness of prolonged-release melatonin in primary insomnia patients with a regular sleep-wake cycle. Sleep Med Res. (2016) 7:16–20. doi: 10.17241/smr.2016.00017

[14] Tsiaka T Kritsi E Tsiantas K Christodoulou P Sinanoglou VJ Zoumpoulakis P. Design and development of novel nutraceuticals: current trends and methodologies. Forum Nutr. (2022) 2:71–90. doi: 10.3390/nutraceuticals2020006

[15] Lengyel M Kállai-Szabó N Antal V Laki AJ Antal I. Microparticles, microspheres, and microcapsules for advanced drug delivery. Sci Pharm. (2019) 87:20. doi: 10.3390/scipharm87030020

[16] Komersová A Svoboda R Skalická B Bartoš M Šnejdrová E Mužíková J . Matrix tablets based on chitosan–carrageenan polyelectrolyte complex: unique matrices for drug targeting in the intestine. Pharmaceuticals. (2022) 15:980. doi: 10.3390/ph15080980, PMID: 36015128 PMC9412913

[17] Zaid AN. A comprehensive review on pharmaceutical film coating: past, present, and future. Drug Des Devel Ther. (2020) 14:4613–23. doi: 10.2147/DDDT.S277439, PMID: 33149558 PMC7605601

[18] Wang Y Xu J Gao N Lv H Sun M Zhang P. Controlled release of bilayer tablet comprising vitamin B_6_ rapid-release layer and melatonin sustained-release layer. Pharm Sci Adv. (2023) 1:100008. doi: 10.1016/j.pscia.2023.100008 PMC12709954 41550780

[19] Abdul S Poddar SS. A flexible technology for modified release of drugs: multi layered tablets. J Control Release. (2004) 97:393–405. doi: 10.1016/S0168-3659(04)00186-5, PMID: 15212872

[20] Chakka G Bindu VH Nischala M. An overview on bilayered tablet technology. J Glob Trends Pharm Sci. (2013) 4:1085. Available at: https://ssrn.com/abstract=3777080

[21] Castán UH Ruiz Martínez MA Rodríguez MTS Ortega Martínez YE Hernández MEM. Bilayer melatonin tablet formulation: a novel approach to therapeutic efficacy. Health Prim Care. (2020) 4:1. doi: 10.15761/hpc.1000192

[22] Crișan AG Porfire A Iurian S Rus LM Lucăcel Ciceo R Turza A. Development of a bilayer tablet by fused deposition modeling as a sustained-release drug delivery system. Pharmaceuticals. (2023) 16:1321. doi: 10.3390/ph16091321, PMID: 37765129 PMC10537489

[23] European Medicines Agency (EMA)/Committee on Herbal Medicinal Products (HMPC). EMA/HMPC, assessment report on *Eschscholzia californica* Cham., herba (2015). Available at: https://www.ema.europa.eu/en/documents/herbal-report/final-assessment-report-eschscholzia-californica-cham-herba_en.pdf

[24] European Medicines Agency (EMA)/Committee on Herbal Medicinal Products (HMPC). EMA/HMPC, herbal medicine: summary for the public, *Melissa officinalis* L., folium. (2013). Available at: https://www.ema.europa.eu/en/documents/herbal-summary/melissa-leaf-summary-public_en.pdf

[25] Draginic N Milosavljevic I Andjic M Jeremic J Nikolic M Sretenovic J . Short-term administration of lemon balm extract ameliorates myocardial ischemia/reperfusion injury: focus on oxidative stress. Pharmaceuticals. (2022) 15:840. doi: 10.3390/ph15070840, PMID: 35890139 PMC9317599

[26] United States Pharmacopeial Convention. Paragraph 731: loss on drying. 10th ed. Rockville, MD: USP Convention (2020).

[27] United States Pharmacopeial Convention. Paragraph 1216: tablet friability. 10th ed. Rockville, MD: USP Convention (2020).

[28] United States Pharmacopeial Convention. Paragraph 1217: tablet breaking force. 10th ed. Rockville, MD: USP Convention (2020).

[29] United States Pharmacopeial Convention. Paragraph 922: water activity. 10th ed. Rockville, MD: (2020).

[30] European Directorate for the Quality of Medicines & Healthcare. European Pharmacopoeia 10.0 In: Paragraph 2.9.1: disintegration of tablets and capsules. Strasbourg: Council of Europe (2019).

[31] European Medicines Agency. Topic Q 1 A (R2) stability testing of new drug substances and products. ICH/2736/99. Available at: https://www.ema.europa.eu/en/documents/scientific-guideline/ich-q-1-r2-stability-testing-new-drug-substances-products-step-5_en.pdf

[32] European Directorate for the Quality of Medicines & Healthcare. European Pharmacopoeia 10.0 In: Paragraph 2.9.3: dissolution test for solid dosage forms. Strasbourg: Council of Europe (2019).

[33] European Directorate for the Quality of Medicines & Healthcare. European Pharmacopoeia 10.0 In: Paragraph 5.17.1: recommendations on dissolution testing. Strasbourg: Council of Europe (2019).

[34] Biorelevant. Media Prep Tool (2023). Available at: https://biorelevant.com/#media_prep_tool_tab

[35] Takagi T Masada T Minami K Kataoka M Izutsu K Matsui K . *In vitro* sensitivity analysis of the gastrointestinal dissolution profile of weakly basic drugs in the stomach-to-intestine fluid changing system: explanation for variable plasma exposure after oral administration. Mol Pharm. (2021) 18:1711–9. doi: 10.1021/acs.molpharmaceut.0c01207, PMID: 33629861

[36] Bassetto R Perin S Amadio E Zanatta S Nenzioni D Bertin W. Safety and efficacy of substance-based medical devices: design of an *in vitro* barrier effect test. Front Drug Saf Regul. (2023) 3:1124873. doi: 10.3389/fdsfr.2023.1124873 PMC12443116 40980104

[37] Biswal B Karna N Bhavsar B. Formulation and evaluation of repaglinide buccal tablet: *ex vivo* bioadhesion study and *ex vivo* permeability study. J Appl Pharm Sci. (2014) 4:96. doi: 10.7324/JAPS.2014.40518

[38] Corti G Maestrelli F Cirri M Furlanetto S Mura P. Development and evaluation of an *in vitro* method for prediction of human drug absorption: I. Assessment of artificial membrane composition. Eur J Pharm Sci. (2006) 27:346–53. doi: 10.1016/j.ejps.2005.11.004, PMID: 16359848

[39] Corti G Maestrelli F Cirri M Zerrouk N Mura P. Development and evaluation of an *in vitro* method for prediction of human drug absorption: II. Demonstration of the method suitability. Eur J Pharm Sci. (2006) 27:354–62. doi: 10.1016/j.ejps.2005.11.005, PMID: 16364612

[40] de Souza Teixeira L Vila Chagas T Alonso A Gonzalez-Alvarez I Bermejo M Polli J . Biomimetic artificial membrane permeability assay over Franz cell apparatus using BCS model drugs. Pharmaceutics. (2020) 12:1–16. doi: 10.3390/pharmaceutics12100988, PMID: 33086670 PMC7589491

[41] Giannola LI De Caro V Giandalia G Siragusa MG D’Angelo M Lo Muzio L . Transbuccal tablets of carbamazepine: formulation, release and absorption pattern. Int J Immunopathol Pharmacol. (2005) 18:21–31. PMID: 16848984

[42] Khan KA Rooke DJ. Effect of disintegrant type upon the relationship between compressional pressure and dissolution efficiency. J Pharm Pharmacol. (1976) 28:633–6. doi: 10.1111/j.2042-7158.1976.tb02816.x, PMID: 11313

[43] Chaturvedi H Garg M Rathore U. Post-compression evaluation parameters for tablets-an overview. Eur J Pharm Med Res. (2022) 4:526–30.

[44] Etcheverry P Grusak MA Fleige LE. Application of *in vitro* bioaccessibility and bioavailability methods for calcium, carotenoids, folate, iron, magnesium, polyphenols, zinc, and vitamins B_6_, B_12_, D, and E. Front Physiol. (2012) 3:317. doi: 10.3389/fphys.2012.00317 22934067 PMC3429087

[45] Takeuchi S Tsume Y Amidon GE Amidon GL. Evaluation of a three compartment *in vitro* gastrointestinal simulator dissolution apparatus to predict *in vivo* dissolution. J Pharm Sci. (2014) 103:3416–22. doi: 10.1002/jps.24112, PMID: 25251982

[46] Manousi N Karavasili C Fatouros DG Tzanavaras PD Zacharis CK. Development and validation of an HPLC-UV method for the dissolution studies of 3D-printed paracetamol formulations in milk-containing simulated gastrointestinal media. Pharmaceuticals. (2022) 15:755. doi: 10.3390/ph15060755, PMID: 35745674 PMC9230883

[47] Kakuk M Farkas D Antal I Kállai-Szabó N. Advances in drug release investigations: trends and developments for dissolution test media. Acta Pharm Hung. (2020) 90:155–69. doi: 10.33892/aph.2020.90.155-169

[48] Marroum PJ. History and evolution of the dissolution test. Dissolution Technol. (2014) 21:11–6. doi: 10.14227/DT210314P11

[49] Dokoumetzidis A Macheras P. A century of dissolution research: from Noyes and Whitney to the biopharmaceutics classification system. Int J Pharm. (2006) 321:1–11. doi: 10.1016/j.ijpharm.2006.07.011, PMID: 16920290

[50] Nathi R Venkata N Prasad D Prasad L Krishna S Konduru N . Flurbiprofen cataplasms: development and validation of *in-vitro* dissolution methods and evaluation of multimedia dissolution profiles. Pharm Sci Adv. (2023) 1:100018. doi: 10.1016/j.pscia.2023.100018 PMC12709885 41550782

[51] Lyu W Omar T Patel H Rodriguez D Ferruzzi MG Pasinetti GM . Dissolution study on grape polyphenol hard gelatin capsule dietary supplements. Front Nutr. (2021) 8:780260. doi: 10.3389/fnut.2021.780260, PMID: 34901128 PMC8656703

[52] Timergalieva VR Gennari CGM Cilurzo F Selmin F Moustafine RI. Comparative evaluation of metformin and metronidazole release from oral lyophilisates with different methods. Sci Pharm. (2023) 91:23. doi: 10.3390/scipharm91020023

[53] Nurhikmah W Sumirtapura YC Pamudji JS. Dissolution profile of mefenamic acid solid dosage forms in two compendial and biorelevant (FaSSIF) media. Sci Pharm. (2016) 84:181–90. doi: 10.3797/scipharm.ISP.2015.09, PMID: 27110508 PMC4839260

[54] Tsume Y Langguth P Garcia-Arieta A Amidon GL. In silico prediction of drug dissolution and absorption with variation in intestinal pH for BCS class II weak acid drugs: ibuprofen and ketoprofen. Biopharm Drug Dispos. (2012) 33:366–77. doi: 10.1002/bdd.1800, PMID: 22815122 PMC3466597

[55] Bai G Wang Y Armenante PM. Velocity profiles and shear strain rate variability in the USP dissolution testing apparatus 2 at different impeller agitation speeds. Int J Pharm. (2011) 403:1–14. doi: 10.1016/j.ijpharm.2010.09.022, PMID: 20883758

[56] Azarmi S Roa W Löbenberg R. Current perspectives in dissolution testing of conventional and novel dosage forms. Int J Pharm. (2007) 328:12–21. doi: 10.1016/j.ijpharm.2006.10.001, PMID: 17084051

[57] Elsayed EW El-Ashmawy AA Mursi NM Emara LH. Biorelevant dissolution testing of numerically optimized multiparticulate drug delivery systems of gliclazide. Dissolution Technol. (2023) 30:88–99. doi: 10.14227/DT300223P88

[58] Wang Z Lou H Dening TJ Hageman MJ. Biorelevant dissolution method considerations for the appropriate evaluation of amorphous solid dispersions: are two stages necessary? J Pharm Sci. (2023) 112:1089–107. doi: 10.1016/j.xphs.2022.12.008, PMID: 36529266

[59] Akter S Addepalli R Netzel M Tinggi U Fletcher M Sultanbawa Y . *In vitro* bioaccessibility and intestinal absorption of selected bioactive compounds in *Terminalia ferdinandiana*. Front Nutr. (2022) 8:818195. doi: 10.3389/fnut.2021.818195, PMID: 35155530 PMC8828953

[60] Dima C Assadpour E Dima S Jafari SM. Bioavailability and bioaccessibility of food bioactive compounds; overview and assessment by *in vitro* methods. Compr Rev Food Sci Food Saf. (2020) 19:2862–84. doi: 10.1111/1541-4337.12623 33337033

[61] Balimane PV Chong S Morrison RA. Current methodologies used for evaluation of intestinal permeability and absorption. J Pharmacol Toxicol Methods. (2000) 44:301–12. doi: 10.1016/S1056-8719(00)00113-1 11274897

[62] Shah B Dong X. Design and evaluation of two-step biorelevant dissolution methods for docetaxel oral formulations. AAPS PharmSciTech. (2022) 23:113. doi: 10.1208/s12249-022-02256-2, PMID: 35441281 PMC9153762

