# Supplementary material for: Designing an effective dissolution test for bilayer tablets tailored for optimal melatonin release in sleep disorder management
Source: Front Nutr. 2024 May 6;11:1394330. doi: 10.3389/fnut.2024.1394330 (PMC11102985; doi:10.3389/fnut.2024.1394330)
Supplement: Supplementary file 1 [file Data_Sheet_1.PDF]

Designing an Effective Dissolution Test for Bilayer Tablets Tailored for Optimal Melatonin Release in Sleep Disorder Management

Rebecca Bassetto, Emanuele Amadio*, Francesco Ciampanelli*, Stefano Perin, Pietro Ilari, Paolo Gaballo, Martina Callegari, Sara Feltrin, Jacopo Gobbo, Samuele Zanatta, Walter Bertin

Table 1S. Dissolution data for ML-Tablet using EU Pharmacopoeia.

Time (h)	Average value	Standard Deviation
0.00	0.0%	0.0%
0.25	68.5%	1.5%
0.50	90.9%	1.4%
1.00	95.9%	0.9%
1.50	97.4%	0.5%
2.00	98.4%	1.0%
2.25	98.3%	1.8%
2.50	98.0%	1.1%
3.00	98.6%	0.7%
4.00	100.0%	2.3%
6.00	100.0%	0.5%
8.00	100.0%	0.9%

Table 2S. Dissolution data for ML-Tablet using SIFC System.

Time (h)	Average value	Standard Deviation
0.00	0.0%	0.0%
0.02	19.2%	2.3%
0.25	75.0%	1.2%
0.50	95.6%	1.9%
1.00	99.5%	0.8%
1.50	100.0%	1.1%
2.00	100.0%	1.3%
2.50	100.0%	0.3%
3.00	100.0%	1.4%
4.00	100.0%	0.9%
6.00	100.9%	2.7%
8.00	100.0%	2.1%

Table 3S. Dissolution data for BL-Tablet using EU Pharmacopoeia.

Time (h)	Average value	Standard Deviation
0.00	0.0%	0.0%
0.25	26.0%	0.3%
0.50	43.0%	2.1%
1.00	66.1%	1.6%
1.50	80.9%	2.8%
2.00	89.6%	2.8%
2.25	97.4%	3.0%
2.50	100.2%	1.9%
3.00	100.0%	1.0%
4.00	100.0%	1.3%
5.00	100.0%	0.5%
6.00	100.0%	1.2%
8.00	100.0%	0.2%

Table 4S. Dissolution data for BL-Tablet using SIFC system.

Time (h)	Average value	Standard Deviation
0.00	0.0%	0.0%
0.02	9.8%	0.4%
0.25	35.1%	1.0%
0.50	46.6%	1.2%
1.00	53.3%	1.1%
1.50	59.4%	0.9%
2.00	64.0%	1.9%
2.30	69.5%	2.9%
3.00	73.0%	1.9%
4.00	77.5%	0.8%
5.00	80.0%	0.3%
6.00	84.2%	3.2%
7.00	91.4%	2.5%
8.00	100.0%	0.7%

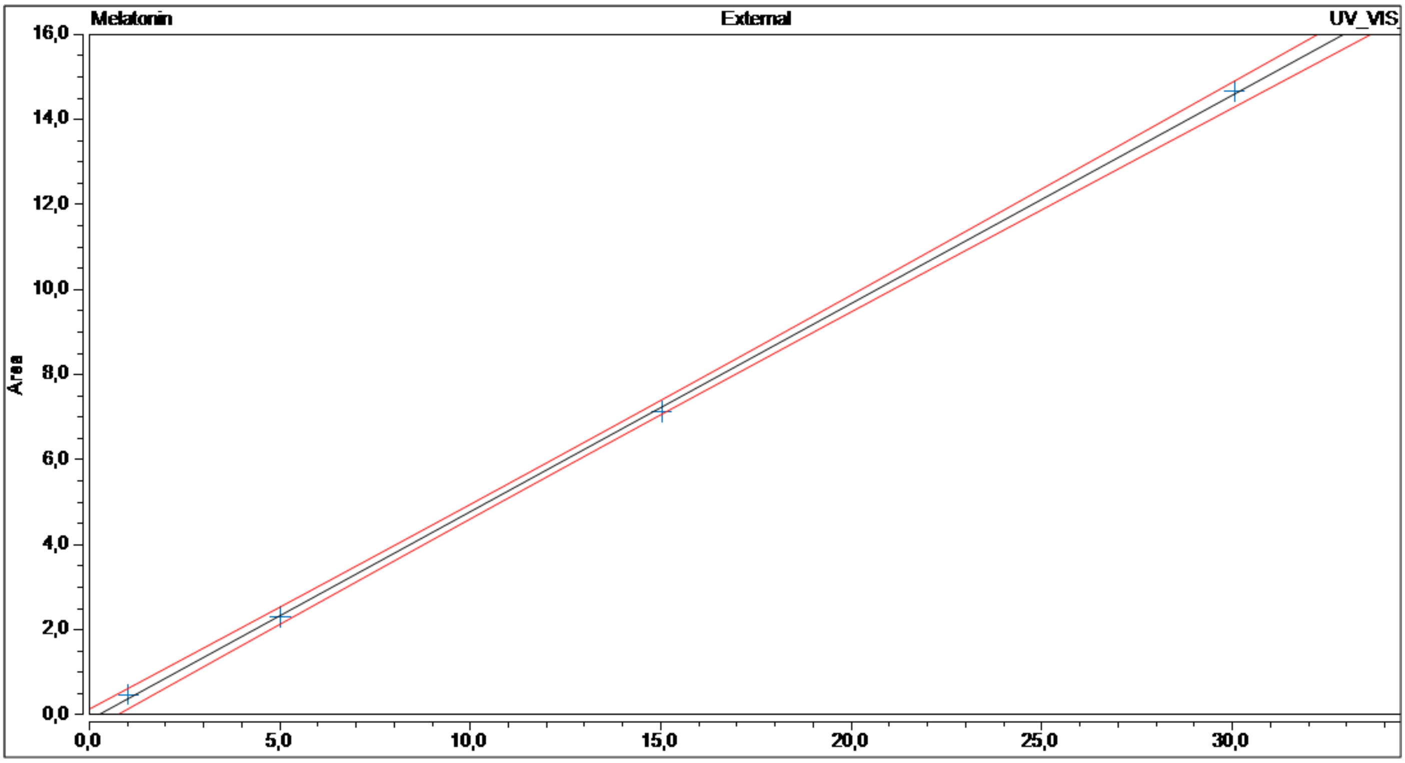

Figure 1S. HPLC-DAD Calibration curve of melatonin.

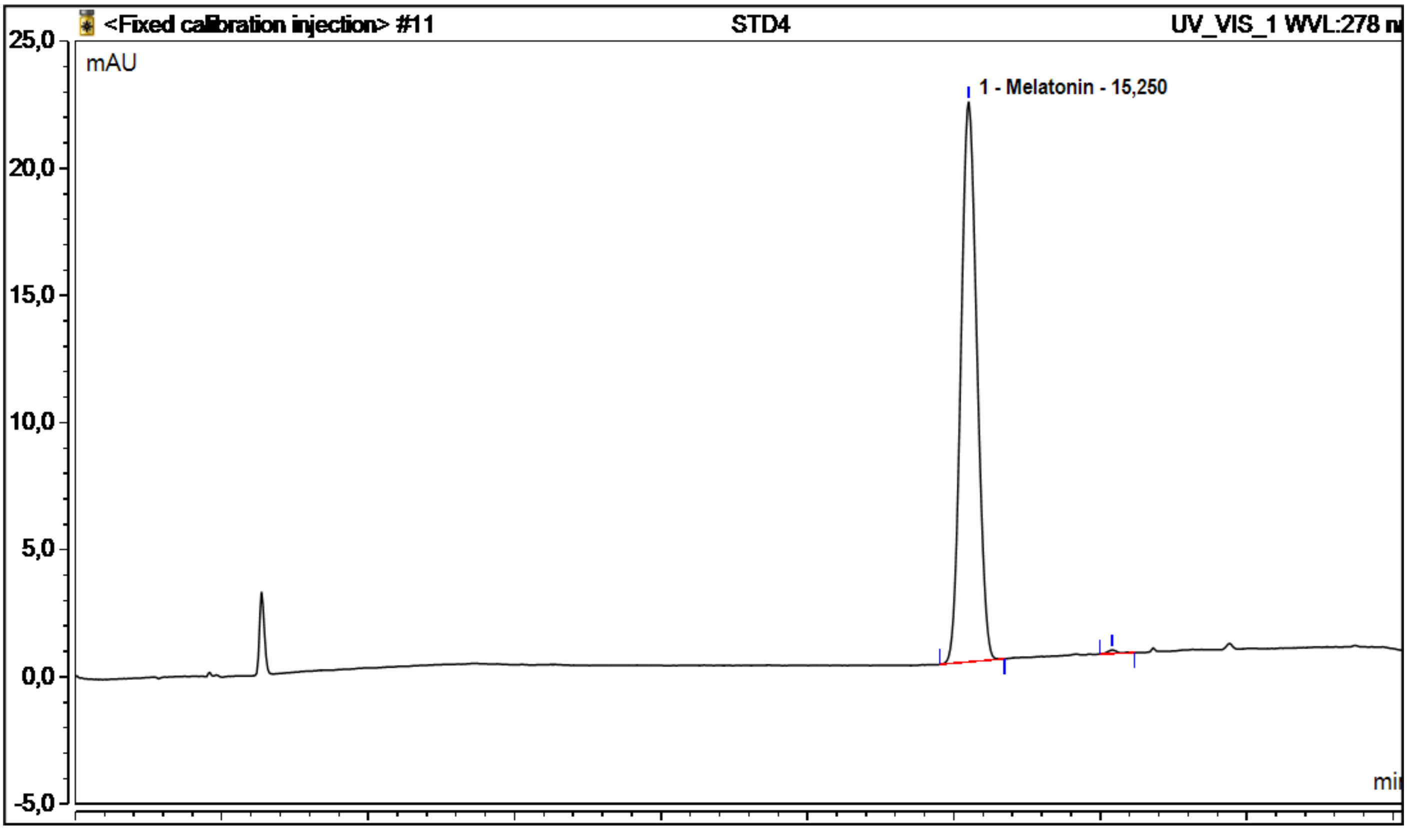

Figure 2S. Typical chromatogram corresponding to a point on the melatonin calibration curve.

Long term zone IVA (30±2°C; 65%±5% UR)
*** backup control, to be performed only if IVB samples are OOS**

Parameter	Specification	T0	C/NC	T6	C/NC	T12	C/NC	T24	C/NC	T36	C/NC
**Physical/organoleptic analysis**											
Appearance	Light blue dotted and white bilayer tablet	compliant	C			compliant	C				
Hardness	20 ± 5 kg	20	C			28	C				
Thikness	7,80 ± 0,20 mm	7,70	C			8,00	C				
Weight, weight uniformity	>1150 mg	1155	C			1170	C				
**Microbiological analysis**											
TAMC	≤ 5 x 10^4 CFU/g-mL					<10	C				
TYMC	≤ 5 x 10^2 CFU/g-mL					<10	C				
Enterobatteriacee	≤ 2 x 10^2 CFU/g-mL					<10	C				
Escherichia coli	Absent/g-mL					absent	C				
Salmonella	Absent/25g-mL					absent	C				
**Chemical analysis**											
Melatonin	1,00 (0,80-1,20) mg/cpr					1,02	C				

Long term zone II (25°C ± 2°C - 60% R.H. ± 5°C)
*** backup control, to be performed only if IVA samples are OOS**

Parameter	Specification	T0	C/NC	T6	C/NC	T12	C/NC	T24	C/NC	T36	C/NC
**Physical/organoleptic analysis**											
Appearance	Orange white and brown trilayer tablet	compliant	C			compliant	C				
Hardness	20 ± 5 kg	20	C			23	C				
Thikness	7,80 ± 0,20 mm	7,70	C			7,80	C				
Weight, weight uniformity	>1150 mg	1155	C			1165	C				
**Microbiological analysis**											
TAMC	≤ 5 x 10^4 CFU/g-mL										
TYMC	≤ 5 x 10^2 CFU/g-mL										
Enterobatteriacee	≤ 2 x 10^2 CFU/g-mL										
Escherichia coli	Absent/g-mL										
Salmonella	Absent/25g-mL										
**Chemical analysis**											
Melatonin	1,00 (0,80-1,20) mg/cpr					1,03	C				

Figure 3S. Stability studies data

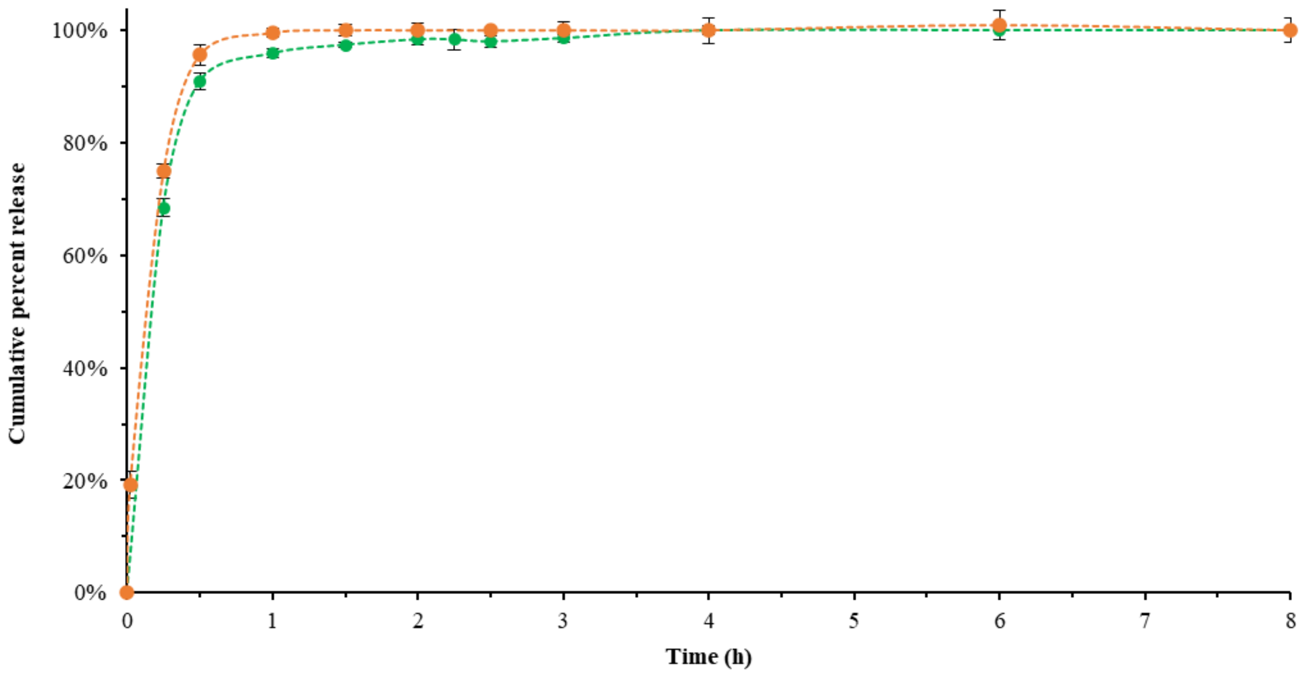

Figure 4S. Comparison of melatonin dissolution in a ML-Tablet between EU Pharmacopoeia (orange) and SIFC-biorelevant (green) methods.

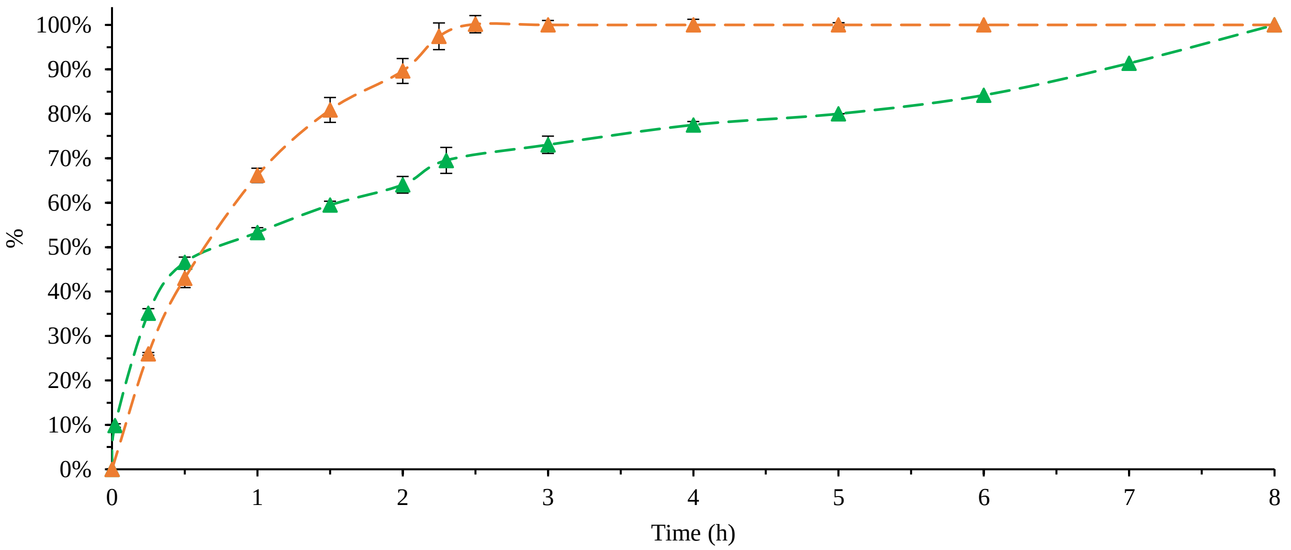

Figure 5S. Comparison of melatonin dissolution in a BL-Tablet between EU Pharmacopoeia (orange) and SIFC-biorelevant (green) methods.

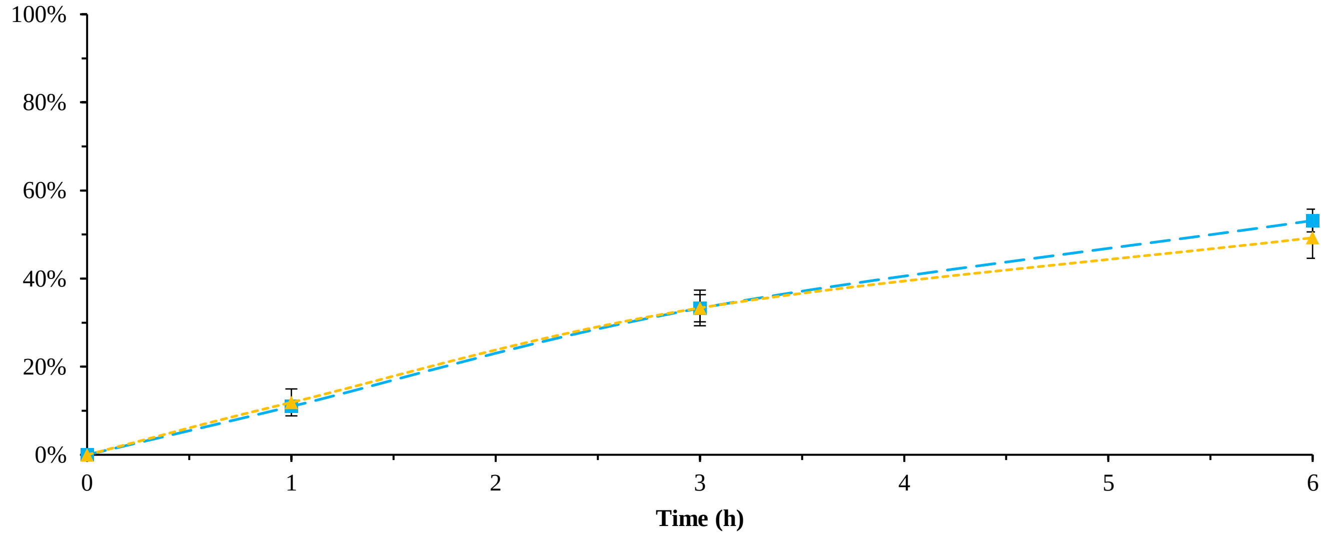

Figure 6S. Comparison between melatonin permeability in BL-Tablet (blue) and ML-Tablet (yellow)

Method development and validation for identification and quantification of Melatonin

- **Materials, Instrument and chromatographic conditions**

Melatonin reference standard was purchased from Merck (Darmstadt, Germany). Water (HPLC grade) and Acetonitrile (HPLC grade) were purchased from Carlo Erba Reagents (Cornaredo, Milan, Italy). All reagents were used without further purification.

The HPLC-DAD analyses were performed on VANQUISH Core/Ultimate 3000 Thermo Fisher Scientific (Waltham, Massachusetts, USA) which include a pump, autosampler, column oven and diode array detector (DAD). The chromatographic separation was carried out on an Acclaim™ C18 column (150 mm x 4.6 mm, 5 mm particle size) from Thermo Fisher Scientific (Waltham, USA) maintained at 30°C. The injection volume was 20 µL. The detection wavelength was set at 278 nm. Mobile phase consisted of A: Water and B: Acetonitrile at flow rate of 1 mL/min. The analytical method for Melatonin was validated using the following elution gradient: 90%-80% A in 4 min, 80% A in 8 min, 80%-50% A in 7min, 50% A in 1 min, 50%-90% A in 1 min, 90% A in 2 min. The data were acquired with Chromeleon 7 Thermo Fisher

Scientific (Waltham, Massachusetts, USA) and processed using Microsoft Excel Microsoft (Redmond, Washington, USA).

- **Stock standard solutions**

Two Stock standard solution of Melatonin Reference Standard were prepared. Stock solution A (SSS_A) was prepared weighing 30.66 mg of Melatonin reference standard into 20 mL (ACN 10% *v/v* in water) volumetric flask. Stock Standard Solution B (SSS_B) was prepared by diluting SSS_A to achieve a concentration of 152,84 μg/mL.

- **Specificity**

The samples for specificity evaluation of HPLC-DAD method consist of: solvent (ACN 10% *v/v* in water), PBS solution, Melatonin reference standard solution, BL tablet solution and ML tablet solution. Each sample was filtered through a 0.22 μm regenerated cellulose syringe filter, and a single injection was performed. No interferences were detected at the characteristic retention time of Melatonin (t_R:15.2 ± 0.4 min).

- **Linearity**

The linearity of the method was evaluated using eight levels of Melatonin standard solutions prepared by diluting a specific amount of SSS_B in the solvent (ACN 10% *v/v* in water) to obtain a concentration range from 0.76 μg/mL to 152.84 μg/mL. Each standard solution was filtered through a 0.22 μm regenerated cellulose syringe filter and injected in triplicate. A linear regression equation of $y = 0.4714x - 0.1777$ was obtained, with a correlation coefficient of 0.9999 (considering a linear R^2 acceptable value of ≥ 0.996).

- **Precision and Accuracy**

The method precision was investigated by analyzing three consecutive injections of a single preparation of Melatonin spiked solution at 100% of the target concentration, and the %RSD values (n=3) of the measured concentrations were used for evaluation. Each solution was filtered through a 0.22 μm regenerated cellulose syringe filter, and the injection was performed. An experimental %RSD value of 0.12 was obtained for the analytical method (considering a precise %RSD acceptable value of ≤ 5.0%).

To assess accuracy, three melatonin reference standard spiked solutions were prepared at 80%, 100%, and 120% of the target concentration, and the %Recovery value of the measured concentrations was used for evaluation. The spiked solutions of each concentration level were prepared individually. Each solution was filtered through a 0.22 μm regenerated cellulose syringe filter and injected in triplicate. A mean %Recovery value of 98.11 was obtained for the analytical method (considering an accurate %Recovery acceptable value of 80% ≤ %Recovery ≤ 120%).
